# Long COVID’s Impact on Patients, Workers, & Society: A review

**DOI:** 10.1097/MD.0000000000037502

**Published:** 2024-03-22

**Authors:** Kevin T. Kavanagh, Lindsay E Cormier, Christine Pontus, Aaron Bergman, Wilmore Webley

**Affiliations:** a Health Watch, Lexington, KY; b Health Watch USA, Eastern Kentucky University, Lexington, KY; c Health Watch USA, Massachusetts Nurses Association, Canton, MA; d Maryland Department of Health, Baltimore, MD; e Department of Microbiology, University of Massachusetts Amherst, Amherst, MA.

**Keywords:** long COVID immune hypofunction diabetes neuropsychiatric misinformation workforce vaccine

## Abstract

The incidence of long COVID in adult survivors of an acute SARS-CoV-2 infection is approximately 11%. Of those afflicted, 26% have difficulty with day-to-day activities. The majority of long COIVD cases occur after mild or asymptomatic acute infection. Children can spread SARS-CoV-2 infections and can also develop long-term neurological, endocrine (type I diabetes), and immunological sequelae. Immunological hypofunction is exemplified by the recent large outbreaks of respiratory syncytial virus and streptococcal infections. Neurological manifestations are associated with anatomical brain damage demonstrated on brain scans and autopsy studies. The prefrontal cortex is particularly susceptible. Common symptoms include brain fog, memory loss, executive dysfunction, and personality changes. The impact on society has been profound. Fewer than half of previously employed adults who develop long COVID are working full-time, and 42% of patients reported food insecurity and 20% reported difficulties paying rent. Vaccination not only helps prevent severe COVID-19, but numerous studies have found beneficial effects in preventing and mitigating long COVID. There is also evidence that vaccination after an acute infection can lessen the symptoms of long COVID. Physical and occupational therapy can also help patients regain function, but the approach must be “low and slow.” Too much physical or mental activity can result in post-exertional malaise and set back the recovery process by days or weeks. The complexity of long COVID presentations coupled with rampant organized disinformation, have caused significant segments of the public to ignore sound public health advice. Further research is needed regarding treatment and effective public communication.

**Key point**

- The best way to prevent long COVID is to not develop COVID-19.
- Neurological and immunological manifestations of long COVID can have a profound impact on children and adults.
- Neurological sequelae can present as brain fog, cognitive disorders, personality changes, and executive dysfunction.
- Immunological sequelae can present as autoimmune diseases and increase susceptibility to common diseases.
- Approximately 11% of acute SARS-CoV-2 infections develop long COVID of these patients, 26% have difficulty with day-to-day functions.
- Rehabilitation involves a “low and slow” process and post-exertional malaise can occur if physical or mental activities are initiated too quickly.
- Vaccinations even after developing acute COVID-19 can help prevent or mitigate the symptoms of long COVID.

## 1. Introduction

On November 1, 2023, the webinar entitled “Long COVID’s Impact on Patients, Workers & Society” brought together international experts to share information regarding long COVIDs’ etiology, presentation, treatment, and societal impact (Table 1). In addition, the public’s hesitancy in adopting public health strategies along with the inhibiting effects of organized disinformation were discussed.

**Table 1:** Webinar presenters, affiliations, and presentation title.

Presenter	Affiliation	Presentation title
Kevin T. Kavanagh, MD, MS	Health Watch USA (sm)	Webinar Overview and Conclusion
Deborah Birx, MD	Past White House Coronavirus Coordinator	Impact of Long COVID on the United States.
Pam Belluck	New York Times Reporter	How Long COVID is Affecting People's Jobs and Their Needs at Work
Peter H. Hotez, MD, PhD	Dean of the National School of Tropical Medicine and Professor of Pediatrics and Molecular Virology & Microbiology at Baylor College of Medicine.	Global Vaccines and Vaccinations: The Science vs The Anti-science.
Eleni Iasonidou, MD	Pediatrician, Founder of Long Covid Greece and a one of the very first Greek representatives to join Long Covid Kids.	Long Covid: A Pediatrician’s View as a Patient and Doctor
Georgios Pappas, MD, PhD	Physician, Researcher and Advocate. Specializing on zoonoses and preparedness against deliberate and natural outbreaks/epidemics	Combating Disinformation regarding COVID-19 and Long COVID
Jane Thomason, MSPH, CIH	Industrial Hygienist, National Nurses United & California Nurses Association	The impact of Long COVID on Nurses
Greg Vanichkachorn, MD, MPH	Occupational and Aerospace Medicine, Mayo Clinic	Symptoms, Treatment and Rehabilitation of Patients With Long COVID.
Wilmore Webley, PhD	Senior Vice Provost for Equity and Inclusion, Professor Department of Microbiology, University of Massachusetts	The Effectiveness of Vaccines to Prevent Long COVID
Brian T. Walitt, MD MPH	Clinician with the NIH’s National Institute of Neurological Disorders and Stroke	Exploring the Post-Acute Sequelae of COVID-19 at the National Institutes of Health
Joycelyn Elders, MD	Past U.S. Surgeon General	Vaccine Hesitancy, Closing Remarks & Conference Summary

The exact incidence of long COVID is not known. As aptly stated by Dr Greg Vanichkachorn, there is not only a lack of consensus regarding how to define long COVID but there is also a lack of agreement in naming the syndrome.^[1]^ Names include long COVID, long haul covid, post-COVID condition, long coronavirus disease, post-COVID syndrome, and post-acute sequelae of COVID-19. But the syndrome does exist. SARS-CoV-2 affects almost every organ of the body and hence can have a myriad of symptoms.^[2]^ Many of these can be delayed in their presentation.

## 2. Narrative review—conference proceedings

The World Health Organization defines long COVID as symptoms that usually present 3 months or more^[3]^ after the onset of an acute SARS-CoV-2 infection and last for at least 2 months, as the Centers of Disease Control and Prevention (CDC) defines the syndrome as symptoms presenting after 4 or more weeks after a SARS-CoV-2 infection.^[4]^ The incidence of long COVID after infection has been found to be higher with earlier strains of the virus than with Omicron.^[5]^ However, due to Omicron’s high infectivity, the prevalence in the general population may be higher. According to the CDC’s COVID-19 Emergency Response Team, in early June of 2022, the prevalence of long COVID in adults with previous COVID-19 was reported to be 18%, but decreased to 11% by January of 2023. The prevalence then stabilized, remaining at 11% in June of 2023.^[6]^ During the BA.4/BA.5 surge, the incidence of long COVID was lowest in those aged 65 years and older (14.8%) and highest in those aged 35 to 44 years (27.6%).^[7]^ An alarming meta-analysis published in *Nature*, found a 48.6% incidence of long COVID in African SARS-CoV-2 patients with a cumulative incidence of post-traumatic stress disorder of 25%.^[8]^ 26.4% of adults with long COVID report significant limitations in their activity.^[6]^ Over half of Canadians (58.2%) with long COVID, have ongoing symptoms, 79.3% for 6 months or more and 42.2% for more than 1 year.^[9]^ Two years after infection, Fernandez-de-Las-Penas et al^[10]^ found post-COVID symptoms in 30% of COVID-19 survivors. Fatigue was the most common symptom (28.0%), followed by cognitive impairments (27.6%), sleep problems (20.9%), depressive levels (18.0%), anxiety (13.4%), and pain (8.4%).

A meta-analysis by Notarte et al^[11]^ found that long COVID is also more commonly found in females and in those with diabetes, pulmonary disease, and organ transplants. Surprisingly, the analysis did not find an association between old age and long COVID.

As of July 2022, Robertson et al^[12]^ reported that 7.3% of 3042 surveyed US adults in the general population reported having long COVID.^[12]^ This would extrapolate to 18.5 million US adults living with long COVID. An online survey by Moy et al found that patients with moderate and severe symptoms (hospitalization may be needed)^[13]^ are 73% to 107% more likely to develop long COVID than patients with mild infections, but 56% of all cases of long COVID were preceded by a mild or asymptomatic infection.^[14]^ Similarly, the Mayo Clinic reported that 75% of all long COVID-19 patients had a mild (not hospitalized) or asymptomatic disease. A study from the Veterans Health Administration found that reinfections also increase the risks of developing long COVID.^[15]^ After 3 COVID-19 infections, Statistics Canada reports a 33.9% incidence of long-term symptoms.^[9]^

Long COVID is a multisystem disease.^[16]^ Symptoms can occur during the acute, medium COVID, and long COVID phases. Some symptoms are due to damage during the acute phase of the infection. However, some organ damages may not present until after 3 months, this includes type I diabetes,^[17–19]^ vascular thrombosis,^[17,19,20]^ thromboembolism,^[19,20]^ autoimmune disease,^[17–19]^ gastrointestinal (GI) problems^[1,17,19]^ along with infections from immune dysfunction.^[18,19,21]^ Often the patient may not associate these delayed sequelae with their acute COVID-19 infection.

An online survey of patients who had an acute SARS-CoV-2 infection revealed that after 2 years fatigue, amnesia, and concentration difficulties were present in 34.8%, 30.3%, and 24.2% of respondents, respectively.^[22]^ Of long COVID patients seeking treatment at Mayo Clinic’s COVID Activity Rehabilitation Program, 80% of patients complained of fatigue, 59% neurological difficulties, 59% respiratory difficulties 45% cognitive impairment, and 26% had mental health symptoms.^[1]^ Early in the pandemic patient led research efforts had similar findings. For example, the Survivor Corps Facebook page listed the 6 most common symptoms as fatigue, muscle and body aches, shortness of breath, difficulty in concentrating or focusing, reduced exercise tolerance, and headaches.^[23]^ Neurological sequelae of long COVID was stressed by 6 of the webinar’s presenters.^[1,17–20,24]^ Fatiguing syndromes should not come as a surprise. This syndrome has been well documented with other infections including Epstein-Barr virus, *Coxiella burnetii* (Q fever), or Ross River virus (epidemic polyarthritis)^[25]^ and viral meningitis.^[26]^

## 3. Etiology of long COVID

Ongoing research has focused on several viable theories regarding the etiology of long COVID which are supported by consistent and strong evidence, these are:

### 3.1. Residual SARS-CoV-2 viruses

SARS-CoV-2 viral particles may persist in a wide array of organs. It is important to note that the cessation of symptoms, lack of viral detection, and even the development of a robust immune response following acute COVID-19 infection, does not always mean the virus has been eradicated. Persistent viral particles can stimulate an immune response resulting in chronic inflammation and local tissue damage. Stein et al^[27]^ studied patients who died with COVID. At autopsy, SARS-CoV-2 RNA was found throughout the body, including brain tissue, for up to 230 days after onset of symptoms. Hany et al^[28]^ recently reported that SARS-CoV-2 can form long-term viral reservoirs in the GI tract’s mucosa.

### 3.2. Reactivation of latent viruses

Many viruses, such as human herpesvirus 6, Epstein–Barr virus (EBV) and cytomegalovirus use latency following an infection as an immune evasion strategy.^[29]^ Recent studies have linked the reactivation of latent viral infections including EBV, human herpesvirus 6 and cytomegalovirus with acute COVID-19 infections.^[29,30]^ It has been estimated that over 90% of the world’s population have been infected at some point by EBV but remain asymptomatic. Reactivation of latent viruses facilitates the entry of SARS-CoV-2 in susceptible cells, increasing viral load and the severity of disease.

### 3.3. Viral superantigens

Hyperinflammatory syndromes, reminiscent of toxic shock syndrome, can occur with COVID-19.^[31]^ Superantigens are immune stimulatory molecules produced by some viruses and bacteria.^[32]^ They commonly elicit a massive systemic immune activation, producing high serum levels of proinflammatory cytokines (i.e., TNFα, IL-1, IL-6, and IFNγ). This type of immune activation can lead to autoimmunity, multi-organ failure and death. Hamdy and Leonardi^[33]^ have shown that SARS-CoV-2 contains a unique superantigen-like peptide not found in any other SARS viruses.

### 3.4. Disruption of the gut microbiome

In humans, the microbiome in the lungs and GI tract resides within the host in a dynamic mutually beneficial relationship, coexisting with the immune system. SARS-CoV-2 can lead to significant dysbiosis of the lung and gut^[34]^ Specifically, Yeoh et al,^[35]^ found that the feces of COVID-19 patients contained a lower number of the bacterial species known to be anti-inflammatory and a higher number of multiple species known to induce an inflammatory response and cause bacteremia. There is growing evidence that this process is associated with the severity of acute COVID disease and long COVID.^[36]^

These varying etiologies are not mutually exclusive and underscores that long COVID is a multi-factorial disease.

## 4. New-onset diabetes in COVID-19

Very early in the course of the pandemic, researchers observed that while diabetes did not increase the risk of SARS-CoV-2 infection, it significantly increased the risks of rapid COVID-19 disease progression and adverse outcomes; and COVID-19 increases the risks of initiation and exacerbation of diabetes.^[37]^ This was a rather surprising bidirectional association, which later progressed to the realization that SARS-CoV-2 infection can induce new-onset diabetes, becoming one of the most significant burdens in long COVID.^[38]^ Diabetes mellitus continues to be a significant metabolic disease around the world, disproportionately affecting African Americans in the United States and leading to significant morbidity and mortality. People who are overweight or obese are at increased risk for diabetes and are simultaneously more likely to have more severe symptoms from COVID-19.^[39]^ SARS-CoV-2 enters cells by binding to the angiotensin converting enzyme 2 receptor with its spike proteins. Angiotensin converting enzyme 2, which is primarily found on the surface of cells lining blood vessels and is ubiquitous throughout the body, including in the pancreas, where it can be found on beta cells in the acini and the islets. SARS-CoV-2 has been shown to infect the beta cells leading to their damage and death.^[40]^ After an initial increase in beta cell mass while the child is a neonate, beta cells enter a replicatory refractory state which “persists throughout life.”^[41]^ Symptoms of diabetes including polyuria, polydipsia, polyphagia, fatigue, weight loss or a combination of these symptoms, typically appear from a few days to 12 weeks after COVID-19 disease onset.^[42]^ As a result, many patients do not initially associate this new-onset diabetes with their COVID-19 infection. This unusual sequelae has been seen in both children and adults and often leads to diabetic ketoacidosis at the time of diagnosis.^[43]^ Recent studies confirm that people who get even mild cases of COVID-19 disease have a significantly greater risk of developing diabetes up to 1 year later.^[38]^ As a result, there is strong evidence of rising diabetes cases in individuals with long COVID.^[44]^ Naveed et al^[45]^ showed in a cohort study, that the risk of new-onset diabetes was significantly higher among people with severe disease compared to those without COVID-19, and male patients were more significantly impacted. In addition, patients diagnosed with prediabetes were at a greater risk of developing diabetes 5 months after acute SARS-CoV-2 infection.^[46]^ It is estimated that 3% to 5% of new onset diabetes over the course of the pandemic can be attributed to SARS-CoV-2.^[45]^

Interestingly, while most of the cases appear to be type 1 diabetes, there have also been many cases of type 2 diabetes associated with post-COVID sequelae.^[47]^ It is therefore possible that a combination of cellular damage and immunological alterations by SARS-CoV-2 contribute to the increased risk of new-onset diabetes in COVID-19 patients suffering from long COVID. A recent study published in *Diabetes Care* found that patients who received COVID-19 vaccination prior to becoming infected with SARS-CoV-2 had a significantly reduced risk of new-onset-diabetes.^[48]^

## 5. Immune hypofunction after acute COVID-19

After the initial waves of SARS-CoV-2, large surges of seemingly unrelated infections started to occur throughout the world. Two competing hypotheses arose to explain this phenomenon, “Immune Debt” versus “Immune Theft” (deficiency). “Immune debt” is a very recent theory which postulates that the immune system has been weakened by lack of exposure to pathogens or vaccines.^[49]^ However, emerging data negate this as a significant factor. There is currently ample evidence that autoimmunity and immune hypofunction play an important role in many patients with long COVID.^[50–53]^ This includes not only the occurrence of Multisystem Inflammatory Syndrome in Children^[54]^ but also an increase in incidence of common infections such as respiratory syncytial virus (RSV) and streptococcal disease.

Mizrahi et al^[55]^ observed that pediatric SARS-CoV-2 patients have a 48% increase in the incidence of streptococcal tonsillitis, 30 to 180 days after infection, and a 34% increase 180 to 360 days after the acute infection. In children ages 5 to 11 years old, there was also a 24% increase in conjunctivitis 30 to 180 days after an acute infection. In 0- to 4-year-olds this increase was 18%. The authors postulated that SARS-CoV-2 may lead to reduced immunity which causes an increased susceptibility to other pathogens. High rates of invasive *Streptococcal* disease have also been reported in Europe,^[56]^ with the United Kingdom being affected the worse.^[57]^ In 2022, over a 12 week period, there were 6600 cases of scarlet fever in England alone.

RSV also surged in 2022. Many supported the hypothesis that this was due to an “Immune Debt” caused by implementation of public health measures. However, this assertion is negated by the observation that large surges were also seen in Sweden,^[58]^ a country which enacted little public health interventions.^[59]^ Finally, a report by Wang et al^[60]^ found a significant increase in risk of RSV infections in children who had previous SARS-CoV-2 infections. In their 2022 study of children with an average age of 2.4 years, there was a 49% increase in risk of RSV if the child had a previous SARS-CoV-2 infection compared to those who did not.

## 6. Neuropsychiatric manifestations of long COVID

Numerous studies have demonstrated the ability of SARS-CoV-2 to invade brain tissue via several routes including the olfactory nerves^[61]^ and optic nerves.^[62–64]^ Song et al^[65]^ confirmed the neuroinvasive potential of SARS-CoV-2. The loss of taste and smell was observed early in the pandemic and largely ignored by many. As stated by Dr Deborah Birx,^[19]^ “that is a significant issue and illustrates this ability of SARS-CoV-2 to actually infect and impact the human brain and memory.” SARS-CoV-2 is “very different” from other respiratory diseases and the flu.

There have been numerous reports regarding problems of memory loss, “brain Fog,” along with laboratory data supporting an organic etiology of these symptoms. The New York Times has reported^[66]^ that the percentage of Americans, aged 18 to 44 years, that have “serious difficulty” in remembering, concentrating, or making decisions rose approximately 37% during the pandemic. Additionally, patients who required ventilation during an acute SARS-CoV-2 infection had a lower I.Q. score of more than 7 points, similar to the aging of the brain by >10 years. Decreases in I.Q. were even observed in those with mild disease.^[67]^

A reduction in brain volume has been demonstrated on brain scans of hospitalized and non-hospitalized patients after being infected with SARS-CoV-2. Significant tissue damage was observed in the orbitofrontal cortex, parahippocampal gyrus, and in regions of the brain connected to the primary olfactory cortex.^[68]^ A decrease in brain volume has also been observed in patients with long COVID.^[69]^

There are functional and anatomical connections between the olfactory bulb and the medial prefrontal cortex (part of the frontal lobe).^[70]^ Toniolo et al have described the SARS-CoV-2 virus as directly targeting the frontal lobes.^[71]^ Changes in the frontal lobes have been demonstrated on EEG, along with MRI and Pet Scan imaging.^[71]^ The orbital frontal cortex (part of the prefrontal cortex) is responsible for executive function, determining if aggression is or is not appropriate, and modulates reactive aggression and antisocial behavior.^[72]^

Executive dysfunction and attention deficits can occur with even mild illness,^[73]^ which may explain the observation of increased traffic accidents in those who are vaccine hesitant.^[74]^ Long COVID has also been reported to be associated with moderate to severe cognitive slowing and prolonged reaction times in 53.5% of studied patients, which can also predispose to accidents.^[75]^

Researchers have observed low serotonin levels in patients with long COVID.^[76,77]^ Low levels of serotonin are implicated in mood disorders, depression^[78]^ and impulse aggression,^[79]^ which may also explain the increased risk of suicidal ideation among healthcare workers.^[80]^ Selective serotonin re-uptake inhibitors, for example Prozac, increase serotonin levels and are used to treat depression. This drug class is being studied for the treatment of long COVID.^[81]^

## 7. Impact on children

The narrative that children are virtually immune to SARS-CoV-2 is dangerous misinformation. In December 2020 (last date data were available) elementary, grammar and nursery schools were responsible for 70% of COVID-19 nonmedical outbreaks in Sweden.^[82]^

Although the incidence of hospitalization and deaths are much lower than adults, children can still become extremely sick and die from COVID-19. Between 2021 and 2022, COVID-19 was a leading cause of death in children.^[83]^ What is even more worrisome is that long COVID is not diagnosed in many children. As stated by Dr Ziyad Al Aly, children do not by themselves state, “I have brain fog.”^[84]^ Symptoms have to be asked for and elicited.

The UK’s Office of National Statistics has reported that over 60,000 children are living with long COVID in the United Kingdom.^[18]^ The true incidence of long COVID in children is unknown, ranging from 1.6% to 70% with the most common symptoms being, fatigue, headache, arthro-myalgias, chest tightness or pain, and dyspnea.^[85]^ In a survey taken 90 days after an acute COVID-19 infection, Funk et al^[86]^ found an incidence of pediatric long COVID of 5.8%. Rao et al^[87]^ observed the most common post-covid symptoms in children to be loss of taste and smell, and myocarditis. Dr Eleni Iasonidou^[18]^ presented data from Buonsenso et al^[88]^ who found the most prevalent symptoms in children to be tiredness and weakness (87.1%), headaches (78.6%), stomach pains (75.9%) muscle aches and pains (68.4%), post-exertional malaise 53.7%, rash 52.4% and heart palpitations (40.2%), lack of concentration (over 60%) and difficulty remembering (over 45%).

Children have also been stigmatized at school for having long COVID. They report being embarrassed, valued less by peers, accused of faking symptoms, and viewed negatively by others.^[89]^

## 8. Vaccination impact on long COVID

Patients who have received 2 COVID-19 vaccine doses are less likely than unvaccinated individuals to develop common symptoms of long COVID.^[90]^ Multiple cross-sectional and observational studies have supported the protective effect of COVID-19 vaccination, mitigating hospitalizations, deaths and even long COVID. This research underscores the necessity of vaccination and receiving boosters before developing an acute SARS-CoV-2 infection. Those who were vaccinated after contracting the disease had a degree of protection but at a lower level.^[91]^ A recent study by Nayyerabadi et al^[92]^ has reported a 40% reduction of long COVID symptoms in patients who were vaccinated after developing an acute infection.^[92]^ At least 8 meta-analyses have concluded that vaccines offer a degree of protection against long COVID.^[93–98]^ A recent systematic review and meta-analysis by Marra et al^[98]^ deduced that for individuals who received 3 vaccine doses, there was a 69% efficacy against long COVID. Lundberg-Morris et al^[99]^ has reported a dose response relationship related to the number of vaccine doses received and the chances of not developing long COVID. There was a 21% reduction with receiving 1 vaccine dose, a 59% reduction receiving 2 doses and a 73% reduction after 3 doses.^[99]^

Laboratory evidence also support the beneficial effects of vaccines. Marra et al^[98]^ have observed a significant reduction in the systemic inflammation caused by pro-inflammatory cytokines and chemokines, even in patients who received a single vaccine dose compared to the unvaccinated. This was true even in the Omicron subvariant era.

## 9. Impact: on workforce

Dr Deborah Birx^[19]^ stressed that COVID-19 will impact the workforce by increasing sick day absences, not only from worker illnesses but also their children and elderly dependents. This loss of productivity is exacerbated by protracted illnesses produced by medium and long COVID. Law enforcement and healthcare are 2 of the most heavily impacted economic sectors.

The effects of long COVID are profound. In June 2022, the U.S. Census Bureau reported that 16 million working-aged individuals were living with long COVID. Two to 4 million workers were not working due to long COVID, with an estimated loss of wages of approximately 170 billion dollars a year.^[100]^ Fewer than half of adults with long COVID who were employed before their acute infection are working full-time after their infection.^[101]^ According to worker compensation claims, more than 1 year after contracting the coronavirus, 18% of long COVID patients had still not returned to work.^[102]^

In a National Nurses United December 2022 survey, Jane Thomason reported that 6.43% of nurses had taken 3 or more months off work to recover from COVID-19. Seventy-eight percent of nurses with long COVID had not sought treatment, of those who did, only about half were able to obtain it. Over a third of nurses with long COVID stated it affected their ability to work. The most common symptoms were fatigue (80.7%), memory or concentration problems (51.5%), joint and muscle pain (51.5%) and headaches or migraines (48.8%). 18% stated their symptoms lasted longer than 12 months.^[102]^

There are also sobering societal implications. Belluck^[103]^ presented data from the Urban Institute which found that among long COVID adults, 42% reported food insecurity (twice the rate compared to individuals who were infected but did not develop long COVID), 20% reported difficulty paying their rent or mortgage, and 23% trouble paying utility bills (with 9% having their utilities shut off).

## 10. Treatment of long COVID

Treatment protocols for long COVID are still largely experimental. Dr Greg Vanichkachorn^[1]^ described a multistep rehabilitation strategy at the Mayo Clinic’s COVID-19 Rehabilitation Program. The first steps include psychological support and patient medical evaluations for serious and common long COVID conditions, along with targeted evaluations. Rehabilitation is patient tailored and as mentioned by multiple presenters, including Drs Vanichkachorn,^[1]^ Iasonidou,^[18]^ and Walitt,^[20]^ post exertional malaise is a major problem. Post exertional malaise can occur after mental or physical exertion and can last weeks. As stressed by Dr Vanichkachorn,^[1]^ “Low and Slow” is better than “No Pain No Gain.”

Experimental drug therapies are also available in many major medical centers. The Mayo Clinic is studying Naltrexone, which when given in low doses is an immune modulator.^[104]^ A recent study by Wong et al^[76]^ found that serotonin depletion was found in long COVID patients and is associated with hypercoagulation, impairment of cognition and reduced vagal signaling. This has led to the possibility of using selective serotonin reuptake inhibitors, such as fluoxetine (Prozac), as a potential treatment.^[105]^

Finally, because of the data supporting viral reservoirs as a cause of long COVID in some patients, vaccinations are also used in an attempt to improve patient symptoms. The Mayo clinic observed a statistical reduction in GI symptoms post vaccination. SARS-CoV-2 mRNA has been found in a wide variety of organs in long COVID patients, including the gut. This further supports viral reservoirs as an etiology of long COVID.^[106]^

## 11. Misinformation

Misinformation and organized planned disinformation are greatly inhibiting the prevention of COVID-19 and long COVID. In the private sector, bots combined with artificial intelligence are flooding social media with influential but erroneous information, which can be even more persuasive than that generated by human authors.^[107,108]^ The Washington Post has published the contents of U.S. government leaked documents which describe 100s of thousands of fake social media bots and accounts from Russia’s Main Scientific Research Computing Center. And only approximately 1% of the fake profiles have been detected.^[109]^ A recent study in *JAMA Internal Medicine* describes AI as a weapon of mass destruction.^[110]^ The authors were able to generate 102 distinct disinformation blog articles in just 65 minutes and were able to generate 20 realistic images in <2 minutes.

Dr Georgios Pappas described disinformation campaigns conducted by foreign adversaries, a major one being Russia. He aptly pointed out that this has happened before in the 1980’s when “Operation Infection” disseminated disinformation stating that “HIV was deliberately made by the US.”^[111]^

In the peer-reviewed literature, an unpublished article in Nature concluded that over the past 2 decades more than 400,000 research articles have been published which appear to have been produced by “paper mills.” The authors estimate that this problem affects 1.5% to 2% of all scientific papers. In medicine the rate is 3%.^[112]^ And this problem has been intensified with artificial intelligence and its ability to “churn out fake research.”^[113]^ Unfortunately even retracted anti-vax studies have been reported as being resurrected in “fake” medical journals.^[114]^

## 12. The impact of disinformation

By far the best way to prevent long COVID is not to become infected with SARS-CoV-2. The impact of disinformation on our response to COVID-19 has been disastrous.^[115]^ Early in the pandemic, Reuters reported that Russia had implemented disinformation campaigns which deployed fake news in multiple languages. A European Union document stated that these “confusing and malicious reports” were adversely impacting the European Union’s ability to communicate their pandemic response.^[116]^

Vaccination rates are falling in the United States. As of December 7, 2023, 2 months after the release of the new COVID-19 booster for the XBB.1.5 variant, only 17.2% of adults and 7.7% of children have received the booster.^[117]^ The CDC has reported that for the 2022 to 2023 school year, 3% of kindergarteners were granted a nonmedical vaccine exemption, up from 2.6% in the previous year.^[118]^ This represents the highest exemption rate ever reported. At a rate above 5% there is a risk of infectious disease outbreaks. And as pointed out by Dr Joycelyn Elders,^[21]^ the anti-vax movement is even affecting vaccination rates in dogs with almost 40% of owners questioning their safety, 30% believe they are medically unnecessary, and >20% consider canine vaccines to be ineffective.^[119]^ It is estimated that 70% of animals have to be vaccinated to prevent an outbreak of rabies.

Our response to COVID-19 has become very politicized. As discussed by Dr Peter Hotez,^[120]^ in Texas there were approximately 100,000 COVID-19 deaths. Forty thousand of these were avoidable and due to peoples’ refusal to obtain a COVID-19 vaccine. In the United States, the number of avoidable deaths approximates 200,000 to 300,000.^[121,122]^ Dr Hotez also described the political or partisan national divide and how it has affected the vaccination rates of the population.^[123]^ A disproportionate number of avoidable COVID-19 deaths among people refusing COVID vaccines during the Delta and BA.1 waves in 2021 to 2022 may have occurred in some states or counties based on political leanings or allegiances. Potentially, long COVID will disproportionately affect these same states and counties.

Our response to disinformation has also been hindered by conflicting and sometimes inaccurate messaging by our public leaders. As pointed out by Ambassador Dr Deborah Birx,^[19]^ the efficacy and duration of COVID-19 vaccines were over promised, and this adversely affected the public’s willingness to embrace future public health guidance. Confidence in our public health leaders was further eroded by delays in recognizing aerosolization of SARS-CoV-2.^[19]^ Jane Thomason from National Nurses United discussed additional concerns regarding the CDC proposals of not recommending N95 masks or respirators for all airborne pathogens and no longer recommending negative pressure rooms for SARS-CoV-1, MERS, and SARS-CoV-2 infected patients.^[124]^ This disinformation has no doubt contributed to the erosion in the public trust of science and scientists. According to the Pew Research Center, only 57% of Americans say science has had a mostly positive effect on society. This share is down 16 points since before the start of the coronavirus pandemic.^[125]^

## 13. Conclusion

There is ample epidemiological and laboratory evidence supporting the need to have heightened concern and continued vigilance for COVID-19 infections. The best way to prevent long COVID is to not contract COVID-19. However, if one contracts COVID-19, vaccination prior to infection significantly lowers the risk of developing long COVID.

As stressed by Dr Joycelyn Elders, we must continue to guard against COVID-19 infections and educate individuals regarding the dangers and prevalence of long COVID. But this will not be an easy task. Dr Deborah Birx^[19]^ emphasized that, “The complexity of the (symptom complex) leads people to question whether its long COVID or not.” A similar conundrum existed regarding tobacco usage and its myriad of associated carcinogens and disease presentations. Like SARS-CoV-2, tobacco inflicts damage in multiple organ systems over an extended period of time. Convincing the public that tobacco was harmful or that there was a causative link between bladder cancer, premature aging, and even type 2 diabetes,^[126]^ literally took decades. Epidemiological statistics and laboratory techniques were too confusing for many in the public to grasp and form a causal relationship between tobacco and disease. Nevertheless, public health prevailed. With an abundant amount of hard work and messaging, the same will be true for COVID-19 and long COVID.

## Acknowledgments

Pam Belluck; Deborah Birx, MD; Joycelyn Elders, MD; Eleni Iasonidou, MD, Peter Hotez, MD, PhD for conference presentations and review of the final version of the manuscript.

## Author contributions

**Conceptualization:** Kevin Kavanagh.

**Resources:** Kevin Kavanagh, Lindsay E. Cormier, Christine Pontus, Aaron Bergman, Wilmore Webley.

**Writing—original draft:** Kevin Kavanagh, Aaron Bergman, Wilmore Webley.

**Writing—review & editing:** Kevin Kavanagh, Lindsay E. Cormier, Christine Pontus, Aaron Bergman, Wilmore Webley.

**Software:** Lindsay E. Cormier, Wilmore Webley.
